# Prevention of Oncogenic *Gammaherpesvirinae* (EBV and HHV8) Associated Disease in Solid Organ Transplant Recipients

**DOI:** 10.3389/ti.2023.11856

**Published:** 2023-11-17

**Authors:** Alaa Atamna, Dafna Yahav, Cédric Hirzel

**Affiliations:** ^1^ Infectious Diseases Unit, Rabin Medical Center, Beilinson Hospital, Petah-Tikva, Israel; ^2^ Faculty of Medicine, Tel-Aviv University, Tel-Aviv, Israel; ^3^ Infectious Diseases Unit, Sheba Medical Center, Ramat-Gan, Israel; ^4^ Department of Infectious Diseases, Bern University Hospital, University of Bern, Bern, Switzerland

**Keywords:** human herpes virus 8, Epstein-Barr virus, Kaposi sarcoma, multicentic Castleman disease, primary effusion lymphoma, posttransplant lymphoproliferative disorders

## Abstract

Long-term risk for malignancy is higher among solid organ transplant (SOT) recipients compared to the general population. Four non-hepatitis viruses have been recognized as oncogenic in SOT recipients—EBV, cause of EBV-associated lymphoproliferative diseases; human herpes virus 8 (HHV8), cause of Kaposi sarcoma, primary effusion lymphoma and multicentric Castleman disease; human papilloma virus, cause of squamous cell skin cancers, and Merkel cell polyomavirus, cause of Merkel cell carcinoma. Two of these viruses (EBV and HHV8) belong to the human herpes virus family. In this review, we will discuss key aspects regarding the clinical presentation, diagnosis, treatment, and prevention of diseases in SOT recipients associated with the two herpesviruses.

## Human Herpes Virus 8 in Solid Organ Transplantation

### Introduction

HHV8 is a DNA virus that belongs to the gamma-herpes virus subfamily. It was first discovered in 1994 as the etiologic agent of Kaposi’s sarcoma (KS) [1]. Four types of KS are distinguished: classic-, endemic-, immunosuppression‐associated-, and AIDS-associated KS [2]. Other HHV8 associated neoplastic disorders include primary effusion lymphoma and multicentric Castleman disease [3, 4].

In SOT recipients, KS is ∼200 fold more frequent than the general population, with cumulative incidence of ∼3%–5% in endemic areas, and <1% in non-endemic areas [5, 6]. Post-transplant KS is a consequence of reactivation of latent infection in seropositive recipients, or a primary donor derived infection in seronegative recipients [7].

Non-neoplastic disorders associated with HHV8 are peripheral cytopenias, hemophagocytic syndromes, acute hepatitis, and KS‐associated herpesvirus inflammatory cytokine syndrome (KICS) [8, 9].

### Epidemiology

The seroprevalence of HHV8 depends on the geographic region. African countries have the highest seroprevalence rates (>50%), whereas seroprevalence in Europe, North America, South and East Asia is lower [10–14]. In low seroprevalence regions, men who have sex with men (MSM) are at increased risk [15].

Both sexual and non-sexual transmission (including blood transfusion and organ transplantation) of HHV8 occurs. SOT recipients may be infected either before transplantation and reactivate the virus post-transplantation, or acquire the virus as a donor-derived infection. Primary HHV8 infection post-transplant increase the risk for HHV8-associated disease [16].

### Non-Malignant HHV8 Disorders

HHV8 infection in immunocompetent individuals is generally asymptomatic, although occasionally associated with a febrile rash in children [17]. In immunocompromised individuals, HHV8 infection has been associated with fever, splenomegaly, maculopapular rash, lymphadenopathy and cytopenia [18] and rarely causes systemic disease with multi-organ failure post-transplantation [6]. Bone marrow suppression, with or without hemophagocytosis was linked to donor derived HHV8 infection in the early post-transplant period [19–21], and rare cases of sexually transmitted primary HHV8 infections post-transplantation were associated with hemophagocytosis [22].

### Malignant HHV8 Disorders

#### Post-Transplant Kaposi Sarcoma (PT-KS)

PT-KS is the most commonly encountered HHV8‐related neoplastic disease [23]. PT-KS mostly develops within the first year post-transplantation [6], and may cause skin lesions involving the extremities, the trunk and the oral cavity [6]. Lesions are characterized by red-blue or purple discoloration, representing the vascular nature of the disease [18]. Visceral involvement occurs in ∼10% of PT‐KS cases, with higher rates (up to 50%) in liver transplant recipients and associated with high a mortality [24, 25]. Disseminated disease without skin lesions exists, and lesions may appear at atypical localizations including the tonsils, urinary bladder and liver [26–28]. The disease can be rapidly-progressive, especially in donor derived cases [29]. In addition to primary infection, risk factors for KS in SOT have been described, with the most prominent factor being residence/origin in endemic countries. Other risk factors include (from higher to lower risk) older age, male gender, thoracic transplantation, and use of cyclosporine and antilymphocyte antibody [18, 30, 31].

### Multicentric Castleman Diseases (MCD)

MCD is characterized by B‐cell transformation to plasmablasts, which subsequently infiltrate multiple lymph nodes and distort their architecture. It typically presents with fever, lymphadenopathy, hepatosplenomegaly, and cytopenia [6]. MCD and PT-KS may occur concomitantly in SOT patients [32–34].

### Primary Effusion Lymphoma (PEL)

PEL is a non‐Hodgkin lymphoma that rarely develops after SOT and affects serous body cavities (pleura, pericardium, and peritoneum) [6]. The median time of presentation is 8 years after transplant, with a wide range from 5 months to 28 years [35]. It presents as a body cavity effusion in the absence of tumor masses. The prognosis is dismal [35].

### Kaposi Sarcoma Herpes Virus (KSHV) Inflammatory Cytokine Syndrome (KICS)

KICS is a systemic inflammation that resembles MCD without pathologic findings in lymph nodes. Generally, patients with KICS also have KS and to a lesser extent may have PEL. Patients with KICS have more severe symptoms, and an increased risk of death [36]. Two cases of KICS have been reported in SOT recipients (Table 1) [8, 37].

**TABLE 1:** Case reports of KSHV Inflammatory Cytokine Syndrome (KICS) in solid organ transplant recipients.

Recipient	Donor	Organ	Presentation	Findings	KS
38 years old female, PSC and CKD, HHV8 IgG Negative	42 years old, HHV8 IgG positive	Liver and Kidney	Persistent fever 12 months after transplantation	Severe anemia and worsening of renal function, severe splenomegaly and small-sized generalized lymphadenopathy, HHV8 viral load ∼189,000 copies/mL	No
54 years old male, TOF, HHV8 IgG Negative	39 years old, Eastern Europe, high risk sexual behavior, HIV negative, positive HHV8 IgG	Heart	Persistent fever 11 months after transplantation	Pancytopenia, ↑creatinine, bilateral pleural effusion, generalized lymphadenopathy, HHV8 viral load ∼183,000 copies/mL	yes

CKD, chronic kidney disease; HHV8, human herpesvirus 8; HIV, human immunodeficiency virus; KS, Kaposi sarcoma; KSHV, Kaposi sarcoma Herpes virus; PSC, primary sclerosing cholangitis; TOF, tetralogy of Fallot.

### Screening and Diagnosis of HHV8 Infection

Recent American society of transplant (AST) guidelines on HHV8 provide a weak recommendation for pre-transplant serological screening of donors and recipients in endemic areas in order to stratify the risk for HHV8 associated disease [6]. In non-endemic areas it is suggested to consider screening for at-risk donors and recipients only (i.e., MSM, people living with HIV or who inject drugs), or for immigrants from endemic countries [8].

The rationale behind recommending serologic screening in endemic settings is the increased risk for KS among seropositive kidney transplant recipients as compared to seronegative recipients (23%–28% vs. 0.7%) [38]. In addition, donor derived post-transplant HHV8 transmission from seropositive donors to seronegative recipients has been described. Nevertheless, HHV8 seropositive individuals are not excluded from organ donation [6]. Preventive reduction of immunosuppression has been suggested in D+R− cases [23].

Lack of standardization of serological assays, variable sensitivity and specificity of these tests, and the absence of an algorithm for management according to serologic findings, result in low rates of pre-transplant screening in practice. In a survey including 51 transplant centers, only one-third performed pre-transplant HHV8 serology. High HHV8 seroprevalence (>6% seropositivity), Italian centers, available protocols for post-transplant viral load monitoring, and having had a recent case of HHV8 disease were associated with screening.

In a study that assessed six different serologic HHV8 assays the Biotrin–DiaSorin IFA and ABI IFA showed the highest agreement with a reference standard of ≥2 concordant positive assays [39].

No AST recommendations are available to direct a schedule of blood viral DNA monitoring in D+R− or R+ SOT recipients, beyond a general recommendation for monitoring in these patients [6]. In a recent survey, 41% of centers reported performing HHV8 PCR monitoring post-transplant, with variable indications including symptomatic patients only, risk-based approaches or universal screening [40]. In case of detectable HHV8 DNAemia, most centers reduced the immunosuppression or changed from calcineurin inhibitors (CNI) to m-TOR inhibitors, with or without addition of antivirals [40]. For viremic patients, guidelines suggest immunosuppression reduction or change to mTOR inhibitors. The rationale for the latter is the antiviral and antiangiogenic effects of sirolimus, though no clinical benefit has been demonstrated in studies [6]. Screening for viral DNA in bronchoalveolar fluid has been suggested for lung transplant recipients and is now under investigation (NCT05081141).

Immune monitoring by HHV8 ELISPOT test is used by some centers as adjunct to HHV8 PCR monitoring in high-risk patients [40]. Absent anti-HHV8 cytotoxic T-cell response has been demonstrated in SOT recipients with KS. It has been suggested that ELISPOT may assist in identifying patients at higher risk for developing KS, and if negative, reducing the immunosuppression may be considered [9].

Table 2 provides a proposed approach for screening or HHV8 pre and post-transplant.

**TABLE 2:** Proposed approach for HHV8 screening pre- and post-transplant.

	Serology^a^	PCR^b^	ELISPOT	Physical exam	Management
Pre transplant	Yes	No	No	No	No
Post transplant	Among D+R− repeated serology may indicate seroconversion—no schedule suggested [40]	Among D+R−, PCR once every 2 weeks for 3 months followed by once monthly to complete 2 years- Among R+, PCR once monthly for 2 years [40]	Among D+R− or R+ may assist as adjunctive test to PCR	For D+R−, skin and mucosal surfaces routine examinations [6]	If PCR positive negative → reduction in immunosuppression or change to mTOR inhibitor [6] If ELISPOT negative → consider immunosuppression reduction [9]

^a^ If a screening approach is not implemented, the following signs and symptoms should merit an investigation for HHV8 if no other cause is found: fever, splenomegaly, maculopapular rash, lymphadenopathy and cytopenia.

^b^ There is no gold standard serology assay.

^c^ Quantitative cut-offs for PCR tests are missing; optimal testing frequency and duration of surveillance have not been determined. Whole blood may be more sensitive than plasma, because if inclusion of the cellular component. Screening is not routinely used by the authors of this review.

### Diagnosis of HHV8 Associated Disease

The gold standard for diagnosing KS, MCD and PEL is histopathological examination of tissue. Immunohistochemical staining of HHV8 latency-associated nuclear antigen confirms the diagnosis. Tissue PCR for HHV8 may assist in confirming the diagnosis. There is no established role for peripheral blood PCR in diagnosing KS. Positive PCR supports the diagnosis of KS, however, it may be negative in ∼20% of KS cases [41]. Highest DNAemia levels were reported in MCD, followed by PEL. Hence, PCR may be more sensitive in these cases, and it has been suggested that negative HHV8 PCR may be used to exclude MCD [42]. High HHV8 DNAemia (>10,000 copies/mL) supports the diagnosis of MCD over KS [43]. In patients with PEL, high viral loads have been demonstrated in effusion fluids [42]. Due to limitations of serology discussed above, it is not currently indicated for diagnosis of HHV8 associated disease [44].

Patients with KICS are almost universally DNAemic. The diagnosis of KICS is based on high levels HHV8 DNAemia, exclusion of other possible causes, and possibly detection of HHV8 in involved organs (bone marrow, liver, and others) [9, 36]. A cutoff value of viral load in plasma ≥1,000 copies/mL or ≥100 copies/10^6^ cells in peripheral blood mononuclear cells has been suggested for diagnosis of KICS [36].

### Prevention

(Val)ganciclovir, cidofovir and foscarnet inhibit the replication of human herpes viruses, including HHV8. Among HIV patients, (val)ganciclovir proved to decrease the incidence of KS [45]. However, effectiveness of these drugs as pre-emptive therapy in cases of positive HHV8 PCR has not been demonstrated.

The need for a vaccine to prevent HHV8 associated malignancies in susceptible populations has been recently raised by the National Cancer Institute. Since the HHV8 genome is highly conserved, it is possible that a single vaccine would provide protection worldwide [46].

### Treatment of HHV8 Related Diseases

Treatment of HHV8 associated malignancies and non-malignant conditions in SOT recipients should first include reduction in immunosuppression (RIS) and/or change from CNI to mTOR inhibitors [47, 48]. Older studies demonstrated between 70% and 100% complete response (CR) of KS following a change from cyclosporin to sirolimus, and 20%–50% CR of KS with RIS [5, 38, 49].

In a more recent study, including 145 SOT recipients with KS, immunosuppression reduction with/without switch to mTOR inhibitors, resulted in a response in >80% of patients [5].

Systemic chemotherapy with an anthracycline or paclitaxel is usually required for KS patients with visceral involvement, extensive lymph node or mucocutaneous involvement, and for patients not responding to reduction/change in immunosuppression [6, 9]. Immunomodulatory therapy with interferon-α is avoided in the SOT setting because of the risk for rejection [50]. Specific chemotherapy regimens are routinely used for the management of MCD and PEL, in addition to immunosuppression reduction [9, 45].

Immunological (ELISPOT) and virological (HHV8 PCR) tests are suggested as part of follow up in the management of KS and other HHV8 related diseases [9].

Several antivirals have *in-vitro* activity against HHV8, including ganciclovir, foscarnet, and cidofovir, while acyclovir is not highly active [51]. Recent NIH guidelines for HIV management do not recommend antivirals as part of KS therapy, based on studies showing limited efficacy [45]. For the treatment of PEL, antiviral drugs may be used as a possible adjunctive therapy, with a CIII level of recommendation [45]. For MCD, two retrospective studies demonstrated remissions using ganciclovir as part of the treatment regimen in HIV patients [45]. This is supported by the rationale of lytic HHV8 infection being present in MCD [52]. There is limited data to support the use of anti-IL6 inhibitors for MCD with no recommendation for general use of these drugs for this indication [45]. Adoptive immunotherapy with cytotoxic T-lymphocytes specific for HHV8 could have a therapeutic role, though there is currently no commercial product available [9].

## Epstein-Barr Virus in Solid Organ Transplantation

### Introduction

Epstein–Barr virus (EBV) is a double-stranded DNA virus of the γ-herpesviridae subfamily [53]. The virus was discovered in 1964 from cultured lymphoblasts of Burkitt’s lymphoma biopsies before being identified as the causative agent of mononucleosis in 1968 [54, 55]. EBV was the first known human oncogenic virus and it efficiently transforms human B-lymphocytes [56–58]. Upon infection, EBV establishes life-long latency in memory B-cells [59, 60]. The pathogenesis of EBV-associated oncogenesis is complex and it is related to the ability of the virus to transform and immortalize B-cells and to impede apoptosis of infected cells [53, 61]. EBV is associated with a large spectrum of diseases, including benign diseases (infective mononucleosis, oral hairy leukoplakia), a number of lymphoproliferative disorders (Burkitt’s lymphoma, some Hodgkin lymphomas, EBV-positive diffuse large B-cell lymphomas, natural killer/T-cell lymphoma, nasal type angiocentric lymphomas, chronic active EBV), epithelial cancers (nasopharyngeal carcinoma, some forms of gastric cancer), smooth muscle cell tumors, and diseases related to immune dysfunction (multiple sclerosis, EBV-associated hemophagocytic lymphohistiocytosis) [61, 62].

In SOT patients, EBV is known to play a major role in the development of EBV-positive post-transplant lymphoproliferative disorders (PTLD), one of the most devastating complications of organ transplantation [53, 63, 64].

### Epidemiology

Seroepidemiologic surveys indicate that >90% of adults are infected with EBV [65, 66]. In developed countries, primary EBV infection tends to occur later nowadays as compared to the past [67–69]. In the transplant setting, donor transmitted EBV infection is common in EBV mismatched (donor EBV+/recipient EBV−) patients. Children are more likely to be EBV-negative, and may acquire the virus from the donor organ or by natural infection, putting them at increased risk for post-transplant primary infection.

### EBV Associated Diseases in SOT Recipients

#### Post-Transplant Lymphoproliferative Disorders (PTLD)

Since the first description of five lymphoma cases in kidney transplant recipients (KTR) in 1969, PTLD has been recognized as a serious complication of SOT [70]. PTLD encloses a heterogeneous spectrum of conditions characterized by lymphoproliferation after transplantation. These disorders range from uncomplicated infectious mononucleosis-like pathology to true malignancies [71]. PTLD is categorized according to the World Health Organization (WHO) 2017 classification, based on its histopathological appearance (Table 3) [77]. Additionally, PTLD is classified according to its temporal occurrence: early-onset PTLD arises within the first year post-transplant, whereas late-onset PTLD occurs thereafter [78]. In contrast to late-onset PTLD, most cases of early-onset PTLDs are associated with EBV [72, 79, 80]. While the incidence rate for EBV-positive PTLD is highest early after transplant, the incidence rate of EBV-negative PTLD is low immediately after transplantation and increases after 4–5 years, resulting in a biphasic pattern of overall PTLD occurrence [81, 82].

**TABLE 3:** Overview of post-transplant lymphoproliferative disorders.

WHO 2017 category	EBV-association	Clonality	Frequency	Clinical features
Non-destructive PTLD	∼100% [72, 73]	No	∼5%	Early-onset, Benign
- Plasmatic hyperplasia				
- Infectious mononucleosis-like PTLD				
- Florid follicular hyperplasia				
Polymorphic PTLD	∼90% [72, 73]	Variable	∼10%	Early and late-onset
Monomorphic PTLD	∼50% [72]	Yes	∼80%	Early > late
B-cell neoplasm				
- Diffuse large B-cell lymphoma				
- Burkitt (like) lymphoma				
- Plasmablastic lymphoma				
- Plasmacytoma like lymphoma				
- Others				
T-cell neoplasms	∼20%	Yes	<5%	Late-onset
- Peripheral T-cell lymphoma				
- Others				
Hodgkin/Hodgkin-like lymphoma	∼90% [74]	Yes	<5%	Early and late-onset

Early-onset, within 1 year post-transplant. Late-onset, >1 year post-transplant. EBV, Epstein-Barr virus; PTLD, post-transplant lymphoproliferative disorder; WHO, world health organization.

A major risk factor for development of EBV-positive PTLD is EBV‐seronegativity pre‐transplant (hazard rate 5–18 as compared to EBV-seropositive individuals) [80, 83–87]. However, in liver transplant recipients the association of EBV-seronegativity and PTLD risk is less pronounced [87]. As children are more likely to be EBV-seronegative before transplantation, PTLD is more common in pediatric SOT recipients. Further, the risk is affected by the type of transplanted organ with intestinal transplant recipients (∼18%) being at highest risk for developing PTLD [88, 89], followed by lung (3%–10%), heart (2%–8%), liver (1%–6%), and kidneys (1%–2%) [90]. In the current era, there was no conclusive association between the type of induction therapy and PTLD risk [91, 92]. The contribution of each immunosuppressive agent to PTLD development is unclear, since patients receive multiple agents in different doses at different times [91]. However, concerns regarding the use of belatacept in EBV-seronegative transplant recipient have been raised [93]. It is for this reason that belatacept is contraindicated in patients who are EBV-seronegative or whose EBV serostatus is unknown prior to transplant [94].

The clinical presentation of PTLD is heterogeneous and depends on the type (non-destructive-, polymorphic-, monomorphic-PTLD) and the localization of disease. Non-specific constitutional symptoms such as fever, unintended weight loss, night-sweats, and fatigue are common. Lymphadenopathy, tonsillar hypertrophy, dysfunction of involved organs, or compression of surrounding structures may occur. More than half of cases presents with extranodal involvement [72, 83, 95]. PTLD frequently involves the gastrointestinal tract (20%–30%), the allografts (10%–15%), and the central nervous system (CNS, 5%–20%) [72, 83, 95]. Therefore, not only lymphadenopathy but also gastrointestinal bleeding or ulcers, allograft dysfunction in combination with masses, and focal neurological signs should rise suspicion for PTLD.

#### EBV-Associated Smooth Muscle Cell Tumor (EBV-SMT)

EBV-SMT is an uncommon neoplasm of immunocompromised individuals [96]. The role of EBV in the tumorigenesis is poorly understood. EBV-SMT is thought to be derived from myogenous vascular smooth muscle cells [97]. The clinical presentation of EBV-SMT is unspecific and depends on the localization of the tumor [98]. Biopsies of smooth muscle tumors in SOT recipients should be evaluated with EBV‐encoded small nuclear RNA (EBER) stains, to establish the diagnosis of EBV-SMT [98] and the differential diagnosis should include KS and mycobacterial spindle cell pseudotumor [96].

#### Non-Malignant EBV-Associated Disease After SOT

The features of these EBV manifestations may include infective mononucleosis, oral hairy leukoplakia [99], and end-organ infections such as encephalitis/myelitis [100] or hepatitis [101]. Some of these manifestations may share clinical features of PTLD (e.g., encephalitis vs. CNS PTLD). Therefore, careful evaluation of these cases is warrant.

Due to the overwhelming clinical importance of PTLD, we will focus on aspects related to PTLD in this review.

### Diagnosis of Post-Transplant Lymphoproliferative Disorders (PTLD)

The diagnosis of PTLD is based on the histopathological examination of appropriate tissue biopsies. Assessing the presence of latent EBV infection of affected cells by (preferably) RNA-in-situ-hybridization targeting EBV-encoded small RNAs (EBER) or by immunohistochemistry targeting latent membrane protein 1 (LMP1) is essential for the diagnosis of EBV‐associated PTLD [102]. Preceding to tissue sampling, radiographic imaging is a crucial initial step to come to a tentative diagnosis. The radiographic evaluation is similar to that used in the evaluation of suspected lymphoma in the non-transplant population [103]. A computed tomography scan (neck to pelvis) is the first step in most centers. MRI may be the preferred modality for suspected cerebral PTLD [104]. Positron emission tomography‐computerized tomography has emerged as a useful imaging modality for detecting suspicious lymph nodes and extranodal lesions and may be helpful to identify optimal sites for biopsy [105]. Establishing a PTLD diagnosis can be difficult and occasionally multiple attempts for getting conclusive tissue biopsies are necessary (especially for gastrointestinal PTLD). In SOT recipients with persistent gastrointestinal symptoms, PTLD should be part of the differential diagnosis and endoscopy with biopsy of ulcers/lesions should be performed [106].

Studies evaluating the diagnostic test characteristics of EBV DNAemia measurements for diagnosing EBV-positive PTLD are limited. In summary, EBV DNAemia above a specific threshold has good sensitivity (∼90%) for detecting EBV-positive PTLD but lacks specificity [107–109] and EBV PCR is not useful for detection of EBV-negative PTLD.

### Prevention of EBV Associated Disease in SOT Recipients

#### Monitoring EBV DNAemia With Reduction of Immunosuppression for Prevention of EBV-Positive PTLD

A monitoring strategy of repeated EBV DNAemia measurement with RIS if a certain threshold is reached or if DNAemia is increasing, is applied by many transplant centers [110], especially for high-risk patients (EBV D+/R−) [108, 111–115]. However, the optimal way to apply this strategy remains unclear. This is also related to the inter-laboratory variability of EBV DNAemia measurements, despite previous efforts for harmonizing results by introducing an international standard [116, 117]. In clinical practice, EDTA plasma or whole blood is used for monitoring EBV DNAemia (Table 4). EBV DNAemia levels are higher when determined in whole blood as compared to EDTA plasma [118, 119]. Therefore, the sensitivity for detection of EBV DNAemia is higher when using whole blood. However, the specificity for detection of EBV‐related disease is better when using EDTA plasma samples [120]. The controversy with respect to the preferred sample type for monitoring EBV DNAemia is ongoing. In our opinion, it is more relevant to ensure that the same type of sample is used and that DNAemia is determined in the same laboratory when longitudinally assessing EBV DNAemia, instead of focusing on the discussion about the preferred sample type. Even though there is no evidence from randomized-controlled trials supporting the usefulness of EBV DNAemia monitoring and RIS, there is some evidence from cohort studies supporting this approach [75, 76, 111]. However, the results of these studies have to be interpreted with caution because of using historic controls [75, 76] (problematic because of decreasing PTLD incidence over time, most likely related to less intense immunosuppression in contemporary versus historic cohorts [72, 111, 121] and the lack of statistical power to show differences due to the rarity of the disease [111]). Although it seems to be appealing from a pathophysiological point of view, there is no strong evidence supporting EBV DNAemia monitoring with RIS for prevention of EBV-positive PTLD. Furthermore, no specific cut-off value for EBV DNAemia to guide preemptive therapy is available, with some studies using any positive titer [122] while others using increasing loads (>10-fold or >1 log10 cp/mL) [122].

**TABLE 4:** Proposed approach for EBV screening pre- and post-transplant.

	Serology	PCR	ELISPOT	Physical exam	Management
Pre transplant	Yes	No	No	No	No
Post transplant	Among D+R− repeated serology may indicate seroconversion—in clinical practice, measurement of EBV DNA in peripheral blood has largely replaced serology for the diagnosis of primary EBV infection	Among D+R−, PCR once every 2–4 weeks for 12 months [75, 76] (author’s personal opinion, week evidence)	For research purpose only	Check for lymphadenopathy during routine clinical controls (author’s personal opinion, no evidence)	Reduction in immunosuppression if high EBV DNAemia^a^ (author’s personal opinion, week evidence) Actively search for PTLD if EBV DNAemia is persistently high^b^

^a^ There is no uniformly accepted EBV DNAemia uniform cut-off for reduction in immunosuppression. This is related to the different types of samples (whole blood vs. EDTA plasma) used for EDTA monitoring and the considerable inter-laboratory variations in EBV DNAemia measurements (even when using the WHO standard).

^b^ There is no established EBV DNAemia cut-off (neither DNAemia level nor duration of persistent DNAemia) for triggering radiologic examinations.

### Antiviral Prophylaxis for Prevention of EBV-Positive PTLD

Several antiviral drugs such as (val)acyclovir, (val)ganciclovir, cidofovir, foscarnet and maribavir inhibit lytic EBV replication [123, 124]. However, these drugs have no effect on latent EBV infection. Since primary EBV infection after transplantation is a major PTLD risk factor, reducing donor‐derived EBV transmission may have an impact on PTLD occurrence. A reduction of primary EBV infection was observed in a cohort of EBV seronegative pediatric KTRs on (val)ganciclovir prophylaxis versus no antiviral prophylaxis [125]. In another cohort of EBV mismatched adult KTRs, antiviral prophylaxis for 3–6 months delayed the rate of EBV primary infection at 100 days post-transplant, but the seroconversion rate 12 months post-transplant was identical with and without prophylaxis (72% vs. 74%) [126]. Recent cohort studies did not find a protective effect of antiviral prophylaxis on PTLD occurrence [72, 127]. These findings are consistent with results of a systematic review published in 2017, concluding that antiviral prophylaxis in high-risk EBV-naive patients has no effect on the incidence of PTLD [128].

### Rituximab for Prevention of EBV-Positive PTLD

The preemptive use of rituximab for prevention of PTLD has become a common strategy in EBV viremic hematologic stem-cell transplant (HSCT) [129, 130]. B-cell depletion before or directly after HSCT, has shown to reduce EBV replication [131, 132] and the incidence of EBV-positive PTLD [133–135] in high-risk patients. The potential effect of rituximab on subsequent PTLD development may be attributable to the depletion of CD20^+^ B-cells, which represent the major reservoir for latent EBV infection. The reduced abundance of these cells at risk for malignant transformation might be linked to a lower PTLD risk [72]. Rituximab use is less well established for prevention of EBV-positive PTLD in SOT recipients. A recent multi-center cohort study reported that rituximab given as part of the induction regimen (mostly in ABO-incompatible kidney transplantation) is associated with a decreased risk for PTLD [72]. A single‐center cohort study reported diminished PTLD rates with rituximab use in heart transplant recipients whose EBV DNAemia did not respond to RIS using a historic control group [75]. Similarly, EBV‐mismatched KTRs with persistent EBV DNAemia or symptomatic EBV infection given rituximab simultaneously with RIS were less likely to develop PTLD compared to contemporaneous controls [114].

### Treatment and Prognosis of PTLD

The first therapeutic measure in treatment of PTLD is RIS under close monitoring of the graft function. There are no evidence-based guidelines on how to reduce immunosuppression, but in clinical practice, stopping anti-proliferative agents and dose reduction of the CNI is the common approach [90]. Significant RIS may not be feasible in all cases and is especially difficult to achieve in thoracic organ transplant recipients due to the risk of life-threatening graft rejection [136]. RIS eradicates the majority of non-destructive PTLD cases. However, for polymorphic and monomorphic PTLD the response to RIS alone is often insufficient [137, 138]. A radiologic reassessment is performed two to 4 weeks after RIS, and if a CR is achieved no further treatment is needed.

In the following section, we summarize the treatment options for polymorphic PTLD and monomorphic diffuse large B-cell lymphoma (DLBCL) PTLD. Treatment of non-DLBCL monomorphic PTLD depends on the histologic classification of the respect lymphoma and follows the same chemotherapy regimens as for immunocompetent patients, and will not be reviewed here. Immunochemotherapy for treatment of DLBCL PTLD is associated with significant toxicity and many SOT recipients are not fit for highly intensive regimens [139]. Therefore, sequential and risk-stratified treatments are applied for treatment of CD20^+^ monomorphic DLBCL PTLD. The PTLD-1 [140], the PTLD-1 third amended [141] and PTLD-2 [142] phase 2 trials are landmark studies that established sequential, risk-stratified PTLD treatment modalities. The PTLD-1 study proved the efficacy and safety of a sequential treatment of four cycles rituximab monotherapy followed by four cycles of cyclophosphamide, doxorubicin, vincristine, and prednisone (CHOP) for patients who did not achieve complete remission with RIS [140]. The most favorable outcomes were seen in patients who achieved a response to rituximab alone prior to chemotherapy, indicating that these patients may belong to a “good-risk group”. In consequence, the PTLD-1 third amended trial assessed a risk-stratified protocol: patients who achieved a CR after four doses of rituximab, received consolidation with rituximab alone while those who did not achieve CR were treated with four cycles of R-CHOP [141]. This trial proved that withholding chemotherapy and performing a rituximab consolidation in patients with CR to rituximab alone is safe and associated with less toxicity [141]. The PTLD-1 third amended trial identified two subgroups with poor prognosis: thoracic transplant recipients and patients with an *International Prognostic Index* (IPI) score >2 [141]. The PTLD-2 trial, *inter alia*, assessed treatment escalation (alternating R-CHOP and R-DHOAx- rituximab, dexamethasone, cytarabine, oxaliplatin) in these patients with poor prognosis [142]. However, the number of patients in this subgroup was low (*n* = 9), the outcome was poor and the treatment related toxicity was substantial [142].

For further information about novel, less established PTLD treatment options such as infusion of third-party EBV-specific cytotoxic T-lymphocytes, CAR-T cell therapy, proteasome inhibitors, burton-tyrosine kinase inhibitors, and histone deacetylase inhibitors in combination with antiviral nucleoside analogues we refer to the recent review of Atallah-Yunes et al. [143].

The introduction of rituximab, the administration of sequential risk stratified treatment regimens, and optimized supportive care have improved the outcome for patients with PTLD. In the PTLD-1 trial, the median overall survival was 6.6 years [140]. Patients with a CR to rituximab alone have better prognosis as compared to rituximab non-responders [141] and thoracic transplant recipients show less favorable outcome as compared to non-thoracic transplant recipients [141, 142].
